# Effect of gastrointestinal digestion on the stability and cytotoxicity of conventional and pegylated liposomes encapsulated with stigmasterol and its esters

**DOI:** 10.1038/s41598-025-17473-5

**Published:** 2025-09-01

**Authors:** Magdalena Rudzińska, Anna Grygier, Anna Olejnik, Katarzyna Kowalska, Joanna Igielska-Kalwat, Dominik Kmiecik, Katarzyna Cieślik-Boczula

**Affiliations:** 1 Faculty of Food Science and Nutrition, Poznań University of Life Sciences, Wojska Polskiego 28, Poznań, 60-637 Poland; 2 Faculty of Chemistry, The University of Wrocław, F. Joliot-Curie 14, Wrocław, 50-383 Poland

**Keywords:** Cancer, Cell biology, Health care, Chemistry, Materials science

## Abstract

Conventional and PEGylated liposomes encapsulated with stigmasterol and its esters were prepared, and their stability and cytotoxicity during in vitro gastrointestinal digestion were determined. The release of stigmasterol and its oxidation products were analyzed using GC-FID. The cytotoxicity before and after digestion was assessed using human normal small intestinal HIEC-6 cells and colon mucosa CCD 841CoN cells. Both liposome PEGylation and the chemical structure of the encapsulated compounds affect the stability and cytotoxicity of the liposomes after gastrointestinal digestion. Esterification of stigmasterol had an effect on the encapsulation of stigmasterol in liposomes, especially PEGylated liposomes, but the level of fatty acid saturation had no significant influence. PEGylation of liposomes did not inhibit sterol oxidation, but in fact led to the formation of oxidation derivatives. Gastrointestinal digestion increased the cytotoxicity of liposomes with stigmasterol (L-St), but liposomes encapsulated with stigmasterol esters had lower cytotoxicity than did undigested liposomes. PEGylation of liposomes did not cause an increase in cytotoxicity to the small intestinal or colon mucosa cells. However, it should be highlighted that the cytotoxic effects of the digested PEGylated liposomes were considerable higher in colon CCD 841CoN cells than in small intestinal HIEC-6 cells.

## Introduction

Phytosterols (PS), also referred to as plant sterols, naturally occur along with their esters in vegetable oils and fats, where they make up to 90% of the unsaponifiable fraction. They are often added to food products in large quantities as functional compounds. Stigmasterol, together with β-sitosterol and campesterol, are the most common phytosterols, with a chemical structure similar to that of cholesterol. They play the same role in plants as cholesterol in animal organisms. The main property of phytosterols in humans is to reduce blood cholesterol levels, which they achieve by blocking cholesterol absorption. Epidemiological studies have established that a diet rich in plant sterols protects against cardiovascular diseases (CVD) and may help counter breast, colon, and lung carcinogenic factors^1–3^. Plant sterols have multiple modes of action, including inhibition of cancer cell growth, induction of apoptosis and invasion, and metastasis, including angiogenesis reduction^1,3^. In addition, it has been reported that long-term PS intake prevents the development of nonalcoholic fatty liver disease^4–6^, and PS have also been demonstrated to be potential therapeutic agents against hepatic fibrosis^7^.

The exact level of the absorption of PS by the human body is not established, as only a few studies have been performed^8,9^. Absorption has however been reported to range from 0% in rabbits to 4% in rats; in humans fed 240–320 mg sitosterol, the estimated absorption level ranged from 1.5–5%.^10^ Compared with the cholesterol percentage absorption (50–60%), the absorption level of phytosterols is much lower, and a minimum intake of 2–3 g/day of is necessary to achieve cholesterol lowering effects^11^.

Phytosterols undergo autoxidative degradation, which can occur both inside and outside of the human body, leading to the formation of phytosterol oxidation products (POPs), which are also called oxyphytosterols^12^. The most common pathway of autoxidative degradation is via a free-radical mechanism, including initiation by reactive oxygen species. These compounds are present in food products, though only at very low concentrations. Oxidized phytosterols are formed during food storage and production, and are absorbed in the human serum, where they can also be detected^13^. These compounds are found in dried canola seeds, refined and cold-pressed oils, French fries, spreads enriched with phytosterols, potato chips, infant formulas, coffee, and heated oils^14–18^.

POPs, like cholesterol oxidation products (oxysterols, COPs), show atherogenic properties^13,19^. It has been suggested that dietary POPs may increase the risk of the formation of severe atherosclerotic lesions^20^. The observation has been made that patients with coronary problems usually show elevated POP levels in serum^21^. The cytotoxicity of POPs has also been demonstrated^22^. The inflammatory properties of POPs have been assessed^19,23,24^. POPs also play a role in the formation of reactive oxygen species during cell metabolism and also affect its metabolism^25^. Oxidative degradation products formed from phytosterols increase oxidative stress, deplete glutathione, lead to mitochondrial dysfunction, and elevate caspase activity^26^. It is thus important to develop new strategies to overcome the oxidative degradation of phytosterols. Data in the literature indicate that enriching food products with monounsaturated phytosteryl ester yields the lowest degradation rate^14^. However, this form of sterol protection is insufficient, and further developments in sterol molecules is required to eliminate the formation of compounds detrimental to human health.

The bioavailability of many biologically active compounds in the digestive tract can be enhanced by encapsulating them into liposomes used as direct delivery system^27,28^. Liposomes are a very attractive system for delivering drugs and bioactive substances into the human body, as they provide protection of these compounds from thermal and oxidative degradation as well as from the effects of light, pH, and enzymes. They are widely used in the pharmaceutical industry and their properties have been thoroughly studied and described by many researchers^29^. However, the use of liposomes in food involves research of a much broader type^30^. Our previous study have shown that phytosterols and their esters with fatty acids can be encapsulated with liposomes^31^. During storage and frying tests, stigmasterol encapsulated with liposomes degraded to form oxyphytosterols, dimers, and oligomers. Some of these degradation products have adverse effects on cell cultures^26,32,33^. The health and safety of foods is crucial for consumers and, in the case of sterols, it is prudent to establish a safe form of delivery that will prevent thermo-oxidative degradation and will heighten absorption in the gastrointestinal tract. PEGylated liposomes show many positive properties over conventional liposomes: they have longer effects in the body, better accumulation in cells, reduced toxicity to healthy tissue, and increased stability. They also have some disadvantages: they reduce cellular uptake, they induce the so-called accelerated blood clearance (ABC), they can lead to pseudo-allergies, and they are unevenly distributed in tissues^34–36^. These challenges indicate that further research and optimization is required to improve the safety and efficacy of PEGylated liposomes.

Despite many years of research into the properties of liposomes, results on their cytotoxicity remain limited. Discrepancies arising from different measurement conditions, readouts and controls for characterizing the interaction of liposomes with cells then lead to further challenges. There is a lack of comparison in the literature of the effects of conventional and PEGylated liposomes on normal cells of the gastrointestinal tract. The use of such carriers in food requires very detailed studies. The aim of this work is to investigate the effect of in vitro digestion on the cytotoxicity of liposomes containing stigmasterol and its esters with myristic and oleic acids. Two types of liposomes (conventional and PEGylated) and three types of normal gastrointestinal cells (hepatic, small intestine, and colon) were used.

## Results and discussion

### Release of stigmasterol during digestion

**Fig. 1:**
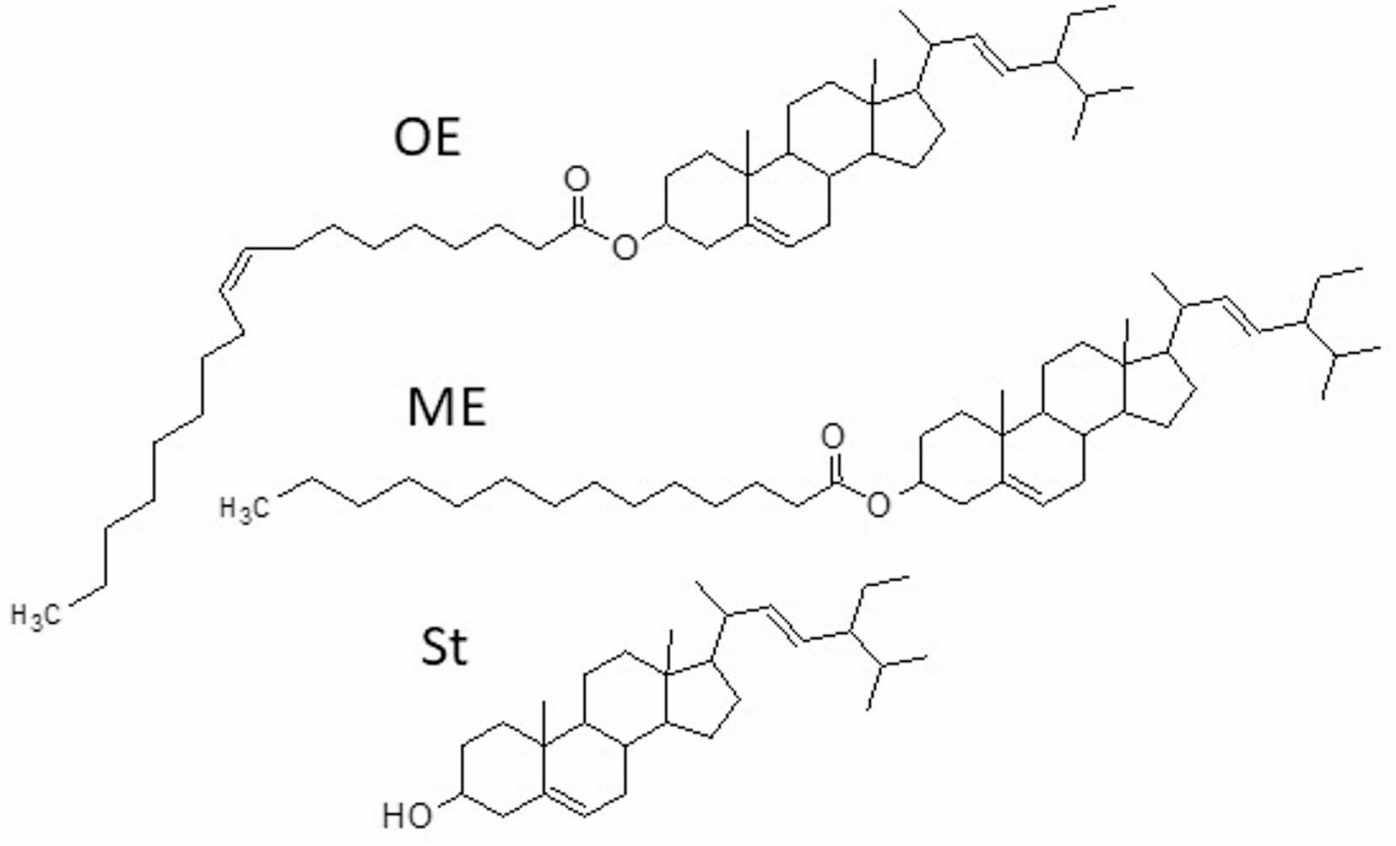
Chemical structures of stigmasterol (St), stigmasterol myristate (ME) and stigmasterol oleate (OE).

**Fig. 2:**
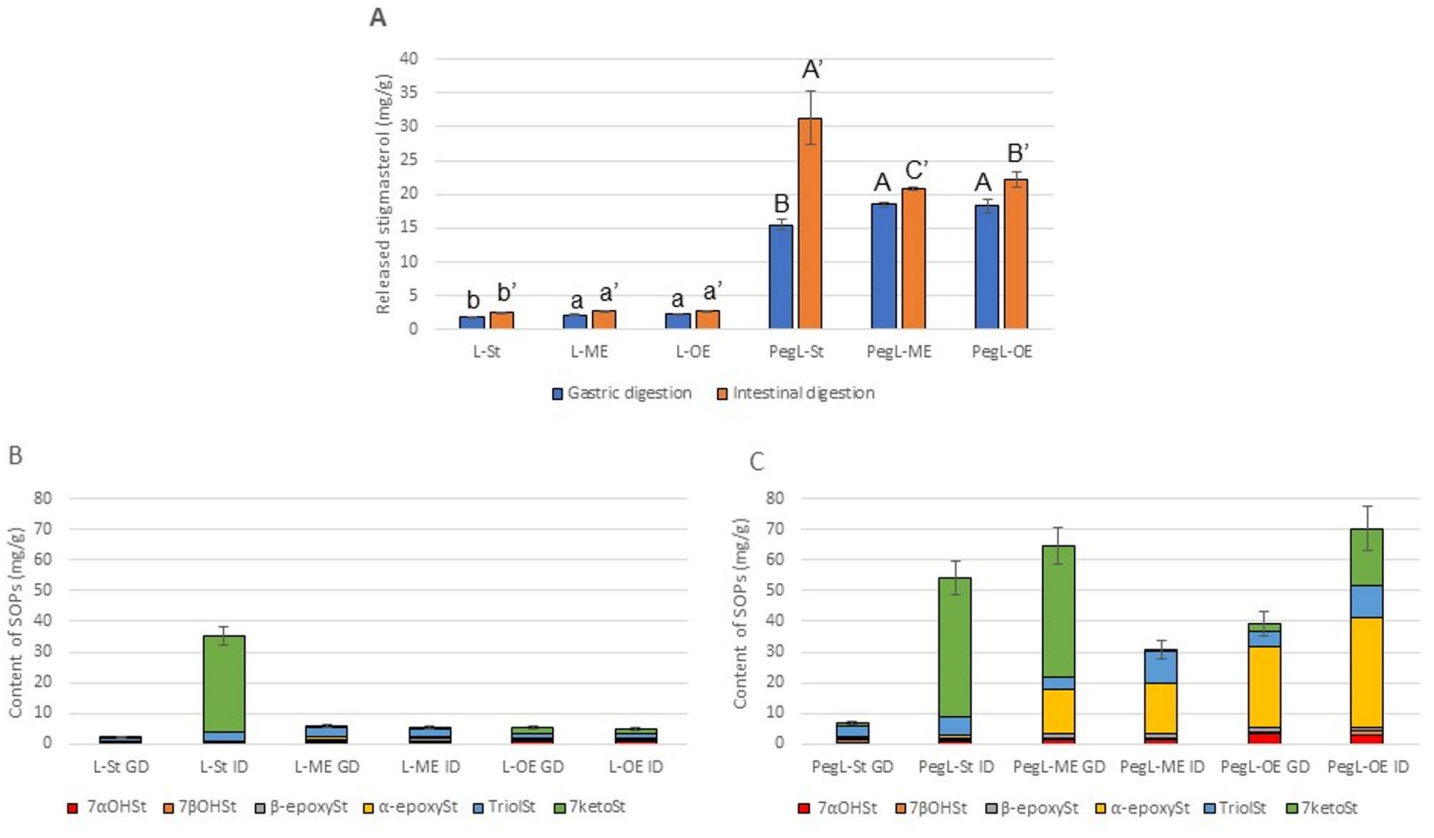
Changes in stigmasterol during digestion. (**A**) stigmasterol released during digestion of conventional and PEGylated liposomes; (**B**) amount and composition of stigmasterol oxidation products formed during digestion of conventional liposomes; (**C**) amount and composition of stigmasterol oxidation products formed during digestion of PEGylated liposomes. GD: gastric digestion; ID: intestinal digestion; St: stigmasterol; ME: stigmasterol myristate; OE: stigmasterol oleate; PegL: PEGylated liposomes. Data are expressed as the mean ± standard deviation of three replicates. Different superscript letters in (A) denote statistically significant differences (*p* < 0.05).

Liposomes encapsulated with free stigmasterol, myristate and oleate esters of stigmasterol (Fig. 1) were treated with enzymes that mimic the digestive processes in the stomach and small intestine. Samples were taken for testing after each step. The experiment was conducted twice and the results are shown in Fig. 2A.

These show that the release of stigmasterol from conventional liposomes is very low, ranging from 1.7 to 2.2 mg/g (0.9–1.3% of encapsulated stigmasterol) after the gastric digestion step and 2.6 to 2.8 mg/g (1.3–1.6%) after the intestinal digestion step.

The level of released stigmasterol was much higher in the case of PEGylated liposomes, ranging from 15.5 to 18.5 mg/g (9.2–12.2%) after the first digestion step and from 20.9 to 31.3 mg/g after the second digestion step, representing 13.6–18.7% of the stigmasterol encapsulated in the liposomes.

The lowest release rate of stigmasterol from the conventional liposomes was found for L-St and the highest rate was seen for L-OE after both digestion steps. The relationship was the same for the PEGylated liposomes after gastric digestion. The slowest release rate for stigmasterol was found in the case of PegL-St, followed in order by PegL-OE and PegL-ME.

The situation was significantly different for PEGylated liposomes after the intestinal digestion step. There was a twofold increase in the release of stigmasterol from PegL-St, whereas the increase in the release rate from PegL-ME and PegL- this increase was significantly smaller.

These results show that the release of stigmasterol from liposomes begins during gastric digestion, especially from PEGylated liposomes. During intestinal digestion, there is a further increase in the release of stigmasterol, but this is only 0.3%-0.4% higher for conventional liposomes than in the case of gastric digestion. Two to nine% more stigmasterol was released than from PEGylated liposomes than at the gastric digestion stage. The greatest amount of stigmasterol was released after the digestion step in the small intestine from PegL-St.

Gastric digestion has little impact on the structure of liposomes. Their diameter quickly decrease and then remains constant, while low pH reduces the electrostatic repulsion between liposomes^37^. However, the structural integrity of liposomal remains virtually unchanged under gastric conditions^38^.

Lipid digestion occurs mainly in the duodenum, which is part of the intestine. The diameter of liposomes increases significantly in the first few minutes after entering the small intestine, and then gradually decreases due to hydrolysis of phospholipids by pancreatic enzymes and the effect of bile salts on liposome components^39^. During this time, the structure of most liposomes is damaged and the release of encapsulated compounds occurs.

Many papers have described the role of cholesterol in stabilizing liposomes and on the optimal lipid: cholesterol ratio for ensuring controlled drug release^40^. However, there are no data in the literature on the stability of cholesterol in liposomes during digestion. In our work, cholesterol was replaced by stigmasterol, a plant sterol. Our results pointing to its stability are innovative and relevant for the use of such liposomes as phytosterol carriers in food.

The release of stigmasterol from liposomes depends on a great many factors. It is influenced by the methods of preparation of liposomes, their composition and structure. In the preparation of liposomes, cholesterol is often added to maintain membrane stability. However, the latest trend is the use of cholesterol analogs derived from plant sterols as stabilizers of liposome membranes. As heart disease risk-lowering substances, liposomes with phytosterols may become a valued food ingredient. While their cholesterol-lowering effects on blood cholesterol are known, their transformation as components of liposomes has not been studied in depth. Cholesterol affects the packing of phospholipid molecules, reduces the permeability of the bilayer, prevents aggregation of liposomes, alters the fluidity of interactions inside the vesicles, makes them more rigid and reduces the efficiency of incorporation of active substances^40^. It plays a key role in strengthening and reducing the fluidity and permeability of the bilayer by increasing its rigidity and strength^41^. Comparing the stability of three sterols (cholesterol, stigmasterol and β-sitosterol) encapsulated in liposomes, there were large differences in their degradation during storage^42^. The chemical structure of the phospholipids used to make the liposomes and the compounds encapsulated in them and the use or nonuse of additional substances such as polyethylene glycol (PEG) have a significant impact on the stability of the encapsulated substances.

Our liposomes were composed of phospholipids (DPPC), free stigmasterol, or its esters, and some of them were additionally PEGylated. All these components had an effect on the physicochemical properties of liposomes, their functionality, and their stability.

The effects of phospholipid composition have been described elsewhere^43^. Analysis has also been performed of the effects of three types of lipids (1,2-dioleoyl-sn-glycero-3-phosphocholine (DOPC), 1,2-distearoyl-sn-glycero-3-phosphocholine (DSPC), and 1,2-distearoyl-sn-glycero-3-phospho-rac-(1-glycerol) (DSPG)), separately and in mixtures, on the physicochemical properties of liposomes, on entrapment efficiency, and on the release of encapsulated substances. DSPG/DSPC showed an increase in the drug’s incorporation efficiency and reduced the particle size distribution. Additionally, PEGylation of these liposomes significantly affected their physicochemical and pharmaceutical properties and showed the most satisfactory characteristics of all the conventional liposomes tested^43^.

It has been widely shown that PEGylation of liposomes affects their pharmaceutical and medical properties. PEGylation of liposomes plays an important role in modifying proteins, peptides, and carriers^44^. PEGylated nanoparticles have a longer circulation time in the blood and consequently accumulate in tumor tissue rather than in normal tissues^45^. Furthermore, PEG modification avoids liposome aggregation, improving the stability of formulations. However, such systems are very complex and need to be investigated for their physicochemical interactions and stability. Although PEGylation has many advantages, conventional liposomes with rigid structures like sphingomyelin/cholesterol liposomes are more suitable for high membrane permeability drugs^44^.

Our results show that PEGylation of liposomes leads to greater release of encapsulated stigmasterol from liposomes during digestion in a model system. Esterification of stigmasterol with fatty acids affects the release of stigmasterol encapsulated in liposomes, especially from PEGylated liposomes. The level of fatty acid unsaturation has no significant effect on this release.

### Formation of stigmasterol oxidation products (SOPs)

Six main stigmasterol oxidation products (SOPs) that form during the gastric and intestinal digestion of conventional and PEGylated liposomes were identified. These were 7α- and 7β-hydroxystigmasterol (7α- and 7βOHSt), α- and β-epoxystigmasterol (α- and β-epoxySt), stigmastentriol (triolSt) and 7-ketostigmasterol (7ketoSt). The data are presented in Fig. 2B and C.

Fewer SOPs were formed during the digestion of conventional liposomes than of PEGylated liposomes. The rate ranged from 2 to 35 mg/g of stigmasterol encapsulated in liposomes after gastric and intestinal digestion, respectively. Of the PEGylated liposomes, the lowest SOP level was seen for PegL-St after gastric digestion and amounted to 7 mg/g, while the highest level of SOPs was detected in PegL-OE after intestinal digestion and was 70 mg/g. In considering the formation of oxyphytosterols in the samples, it is important to take into account their composition as well as their content.

The amount of 7α- and 7βOHSt, like α- and β-epoxySt in conventional liposomes during both digestion stages, was less than 1 mg/g. The increase in total SOPs in sample L-St is mainly associated with the increase in 7ketoSt, which ranged from 0.2 after gastric digestion to 31.7 mg/g after intestinal digestion. 7ketoSt has proinflammatory potential and can cause disturbances in mitochondria or lipid metabolism^46,47^.

The level of triolSt is important: this was highest (at 3 mg/g) for L-ME after both stages of digestion, like L-St after intestinal digestion. The triol-ST level was 1 mg/g for L-OE after both stages of digestion and for L-St after gastric digestion. Data on the toxicity of triolSt are lacking in the literature. By analogy with cholesterol triol (triolCh), it can be assumed that this is the most toxic oxidation derivative of stigmasterol. TriolCh is a highly toxic oxysterol with a broad spectrum of effects, including oxidative stress, mitochondrial damage and pro-inflammatory effects. Due to its toxic properties, it is considered a potential agent in the development of degenerative diseases such as atherosclerosis and cancer^48^.

The SOP composition of PEGylated liposomes is more diverse. The lowest level was found for 7βOHSt, at less than 1 mg/g for all samples, and for β-epoxySt, which ranged from 0.6 mg/g for PegL-St to 1.6 for PegL-ME after both stages of digestion. 7αOHC depended more on the type of compound encapsulated than on the stage of digestion, and was 0.7 mg/g for PegL-St for both stages of digestion. The value for PegL-ME was 1.2 and 1.5 mg/g and that for PegL-OE was 3.1 and 3.5 mg/g after gastric and intestinal digestion, respectively. The level of α-epoxySt was still very low for PegL-St (< 1 mg/g), but in the case of PegL-ME it amounted to 15–16 mg/g after both stages of digestion. PegL-OE showed the highest level of α-epoxySt at 27 and 36 mg/g after gastric and intestinal digestion. The highest level of 7ketoSt of 46 mg/g, similar to the case of L-St, was noted for PegL-St after intestinal digestion. Very high levels of this SOP were also detected in PegL-ME after gastric digestion (43 mg/g), followed by PegL-OE after intestinal digestion (19 mg/g). The triolSt content was greater after intestinal digestion than after gastric digestion and amounted 6, 11, and 11 mg/g for PegL-St, PegL-ME, and PegL-OE, respectively.

The literature lacks data on the formation of sterol oxidation products in liposomes. Our results show that the PEGylation of liposomes did not inhibit sterol oxidation, but can in fact induce formation of SOPs.

### Cytotoxicity of liposomes after digestion

**Fig. 3:**
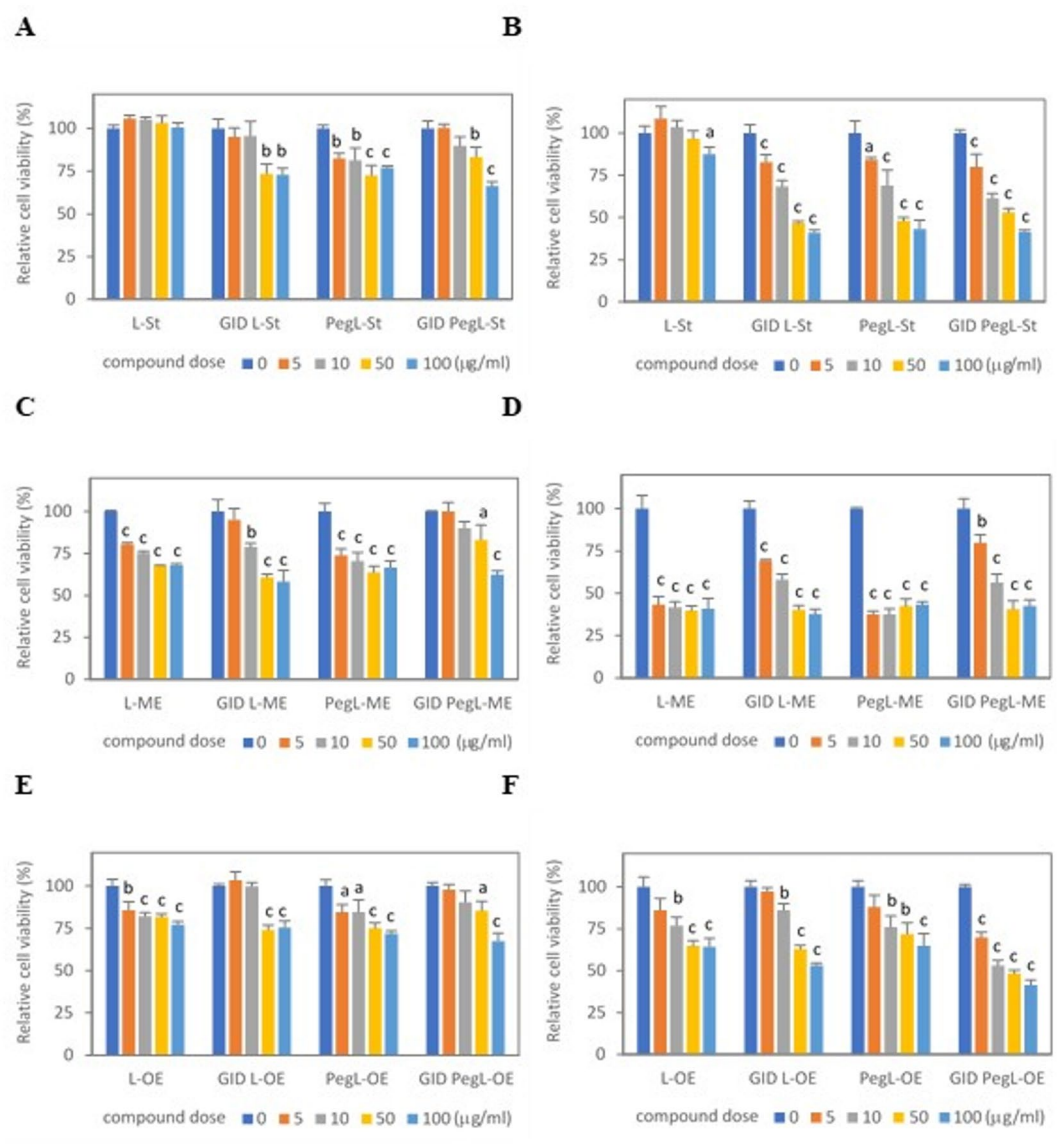
Cytotoxicity of conventional liposomes (L) and PEGylated liposomes (PegL) encapsulated with free stigmasterol (St) (**A**,** B**), stigmasterol mirystate (ME) (**C**,** D**), and stigmasterol oleate (OE) (**E**,** F**), both undigested and gastrointestinally digested (GID), to normal human small intestinal cells (HIEC-6 cells) (**A**,** C**,** E**) and colon mucosa cells (CCD 841CoN cells) (**B**,** D**,** F**). Values represent the means (*n* = 3) ± SD. The post hoc Tukey’s post hoc test determined the significance of cytotoxic effects of liposome concentrations of 5, 10, 50, and 100 µg/ml (^a^*p* ≤ 0.05; ^b^*p* ≤ 0.01; ^c^*p* ≤ 0.001).

We evaluated the cytotoxicity of liposomes encapsulated with free stigmasterol and myristate, as well as oleate stigmasterol esters, in normal human small intestinal epithelial HIEC-6 cells and colon mucosa CCD 841CoN cells. These cells were exposed to the liposomes for 48 h. The cytotoxic effects were assessed using the MTT assay. The data from the MTT analysis are presented in Fig. 3. Based on the results of the MTT assay, the first cytotoxic doses (IC_10_) of the liposomes were calculated and shown in Table 1. The findings indicate that liposomes encapsulated with free stigmasterol (L-St) exhibited the lowest cytotoxicity in both intestinal cell lines (Figs. 3A and B), which results in the highest IC_10_ doses (Table 1). The concentration of L-St that produced a 10% decrease in colon cell viability was found to be 95.0 ± 10.8 µg/ml. In small intestinal cell cultures, no cytotoxic effects were observed after treatment with L-St at concentrations up to 100 µg/ml (Fig. 3A). Liposomes encapsulated with stigmasterol myristate (L-ME) and oleate (L-OE) demonstrated significantly higher cytotoxic potential than liposomes containing unesterified stigmasterol (Fig. 3C, D, E and F). Based on the IC_10_ assessed for both intestinal cell types, the liposomes can be ranked in the following order by cytotoxicity: L-St < L-OE < L-ME (Table 1).

**Table 1:** First cytotoxic doses (IC_10_) of conventional liposomes (L) and pegylated liposomes (PegL) encapsulated with free stigmasterol (St), stigmasterol mirystate (ME), and stigmasterol oleate (OE), both undigested and gastrointestinally digested (GID), determined in normal human small intestinal HIEC-6 and colon mucosa CCD 841CoN cell cultures.

Liposomes	Cytotoxic dose (IC_10_) (µg/ml)	
	HIEC-6 cells	CCD 841CoN cells
L-St	> 100	95.00 ± 10.77
GID L-St	25.40 ± 3.94^***^	3.02 ± 0.48^***^
PegL-St	3.01 ± 0.43^###^	3.38 ± 0.79^###^
GID PegL-St	9.28 ± 0.89^***^	2.80 ± 1.13
L-ME	1.06 ± 0.13	0.31 ± 0.05
GID L-ME	6.30 ± 1.38^**^	1.57 ± 0.52^**^
PegL-ME	0.82 ± 0.10	0.22 ± 0.03
GID PegL-ME	13.21 ± 4.97^**^	2.42 ± 0.47*
L-OE	2.51 ± 0.11	3.84 ± 0.59
GID L-OE	25.39 ± 3.12^***^	8.40 ± 1.42^**^
PegL-OE	3.56 ± 1.11	4.07 ± 0.55
GID PegL-OE	14.26 ± 6.66^**^	1.66 ± 0.21*

Values represent the means (*n* = 3) ± SD. Significant differences: *digested liposomes compared to undigested liposomes (*p ≤ 0.05; **p ≤ 0.01; ***p ≤ 0.001); ^#^PEGylated liposomes compared to non-PEGylated liposomes (^#^*p* ≤ 0.05; ^##^*p* ≤ 0.01; ^###^*p* ≤ 0.001).

Our study has shown that PEGylation of L-St liposomes significantly enhanced their cytotoxic activity against intestinal cells (Figs. 3A and B). The first inhibitory effects of the PEGylated liposomes (PegL-St) were observed at concentrations of 3.01 ± 0.43 µg/ml for IEC-6 cells and 3.38 ± 0.79 µg/ml for CCD 841CoN cells (Table 1). In contrast, PEGylation of liposomes encapsulated with stigmasterol esters (PegL-ME and PegL-OE) did not affect their cytotoxic potential, as indicated by comparable IC_10_ values between conventional (L-ME, L-OE) and PEGylated (PegL-ME, PegL-OE) liposomes (Table 1). PegL-ME exhibited the highest cytotoxicity of the PEGylated liposomes (Fig. 3C and D), with its IC_10_ values significantly lower than those for PegL-St and PegL-OE in both intestinal cell cultures (Table 1). The cytotoxic effects induced in small intestinal cells and colon mucosa cells by PegL-St (Figs. 3A and B) and PegL-OE (Figs. 3E and F) were comparable, as shown by their first cytotoxic doses (Table 1). Their cytotoxicity can be ranked as follows on the basis of the IC_10_ values of PEGylated liposomes assessed in intestinal cell cultures (HIEC-6 and CCD 841CoN cells): PegL-St (3.01 ± 0.43 and 3.38 ± 0.79 µg/ml) = PegL-OE (3.56 ± 1.11 and 4.07 ± 0.55 µg/ml) < PegL-ME (0.82 ± 0.10 and 0.22 ± 0.03 µg/ml).

We here evaluated the changes in the cytotoxic potential of liposomes encapsulated with stigmasterol and its esters having undergone gastrointestinal digestion simulated in the artificial digestive tract. The digestion process in the stomach and small intestine increased the cytotoxicity of the liposomes L-St toward small intestinal IEC-6 cells and colon mucosa CCD 841CoN cells, as was evidenced by significantly lower IC_10_ values. The initial inhibitory concentrations of gastrointestinally digested (GID) L-St were estimated to be 25.40 ± 3.94 µg/ml for HIEC-6 cells and 3.02 ± 0.48 µg/ml for CCD 841CoN cells (Table 1). In contrast, the cytotoxic potential of GID liposomes encapsulated with stigmasterol esters (GID L-ME and GID L-OE) was lower than that of undigested liposomes (L-ME and L-OE).

In contrast, the cytotoxic potential of GID liposomes encapsulated with stigmasterol esters (GID L-ME and GID L-OE) was found to be lower than that of undigested liposomes (L-ME and L-OE). For instance, the IC_10_ values of L-OE and GID L-OE in small intestinal cell cultures were determined to be 2.51 ± 0.11 µg/ml and 25.39 ± 3.12 µg/ml, respectively. Similarly, the IC_10_ inhibitory concentrations for these liposomes in colon cells were 3.84 ± 0.59 µg/ml for L-OE and 8.40 ± 1.42 µg/ml for GID L-OE. A similar trend could also be seen in comparing L-OE and GID L-OE liposomes to both intestinal HIEC-6 and CCD 841CoN cells (Table 1). The liposomes digested in the gastrointestinal tract are here listed by their cytotoxicity to small intestinal cells (using IC_10_ values) as follows: GID L-St (25.40 ± 3.94 µg/ml) = GID L-OE (25.39 ± 3.12 µg/ml) < GID L-ME (6.30 ± 1.38 µg/ml). The ranking of GID liposome cytotoxicity to colon mucosa cells was slightly different: GID L-OE (8.40 ± 1.42 µg/ml) < GID L-St (3.02 ± 0.48 µg/ml) < GID L-ME (1.57 ± 0.52 µg/ml).

Our findings suggest that the PEGylation of liposomes may play a significant role in the cytotoxicity of liposomes encapsulating stigmasterol and its esters, particularly during digestion. In small intestinal cell cultures, the cytotoxic effects of GID PEGylated liposomes were considerably lower than those of the PEGylated liposomes that had not undergone digestion in the gastrointestinal tract. However, digested PEGylated liposomes led to a greater reduction in small intestinal cell viability than did digested non-PEGylated liposomes, regardless of the type of stigmasterol derivative that was encapsulated.

A comparison of the first cytotoxic doses of GID PEGylated liposomes encapsulated with stigmasterol and its esters did not show any significant differences in cytotoxicity to the small intestinal cells. A similar trend was seen when colon mucosa cells were treated with digested PEGylated liposomes. However, it should be noted that the cytotoxic effects of GID PEGylated liposomes were considerably higher in colon CCD 841CoN cells than in small intestinal HIEC-6 cells (Table 1).

Liposomes are very often used in medicine and cosmetics. Their use in food as carriers of bioactive compounds is still rare. There are some examples in the literature^27,49,50^ showing that PEGylated liposomes are stable and can significantly suppress the degradation of biologically active molecules encapsulated in them in intestinal fluid. The results showed for insulin-encapsulated in PEG-liposomes^49^ demonstrated that surface coating liposomes with PEG gained resistance against digestion by bile salts and increased the stability in the GI tract. Since PEG molecules are well hydrated in an aqueous solution, a thick water layer forms on the surface of PEG-liposomes, preventing direct interaction between bile salts and the lipid membrane^49^. In oral delivery, the PEGylation of DPPC liposomes significantly suppresses the enzymatic degradation of recombinant human epithelial growth factor (rhEGF) encapsulated in PEG-liposomes^50^. Furthermore, PEGylated coating materials for liposomes are recognized as an effective inhibitor of the P-glycoprotein efflux system, thereby enhancing the overall bioavailability of encapsulated biologically active molecules^51^. In summary, PEGylation, a technique originally developed to extend the half-life of drugs in the blood, is now widely used in the oral delivery of liposomes^27^.

## Materials and methods

### Materials

Standards of stigmasterol (≥ 95%), myristic acid (≥ 98.0%), oleic acid (≥ 99%), cholesteryl oleate (98%), 5α-cholestane, heptadecanoic acid methyl ester, native pepsin from porcine stomach mucosa, lipase from porcine pancreas, bile salts, salts for preparing electrolytes, sodium hydroxide, anhydrous pyridine, the catalysts dicyclohexylcarbodiimide (DCC) and 4-dimethylaminopyridine (DMAP), high-purity silica gel 70–230 mesh, PEG2000 PE, and all solvents were purchased from Sigma-Aldrich (St. Louis, MO, USA). The internal standard 19-hydroxycholesterol was purchased from Steraloids (Newport, RI, USA). The silylation mixture of BSTFA [*N*,*O*-Bis(trimethylsilyl) trifluoroacetamide] with 1% TMCS (trimethylchlorosilane) was obtained from Fluka Chemie, while the SEP-PAK amino cartridges were sourced from Waters (Milford, USA). Dipalmitoylphosphatidylcholine (DPPC) was purchased from Avanti Polar Lipids (Birmingham, AL, USA).

### Esterification

Chemical esterification was used to obtain the stigmasteryl esters with myristic and oleic acids (Neises & Steglich, 1978; Raczyk et al., 2017). Briefly, stigmasterol was dissolved in dichloromethane, and the catalysts (500 mg DCC and 15 mg DMAP) and fatty acids were added. Esterification was run in argon at room temperature for 24 h in the dark. Distilled water was added to the finished reaction, and the mixture was separated in a separatory funnel. The lower layer was collected and the cleaning procedure was repeated three times. The solvent was evaporated and the residue dissolved in hexane. The resulting mixture was cleaned using a silica gel column and the purity was verified by TLC in comparison with the cholesteryl ester standard. The quality of the esterified esters was determined using 1 H-NMR and GC–MS. The chemical structures of stigmasterol and its esters are presented in Fig. 1.

### Preparation of liposomes

#### Conventional liposomes

A chloroform–methanol solution of DPPC and stigmasterol or its esters were dried under a stream of nitrogen^52^. The dry lipid films were hydrated with the addition of 1 mL of buffer at a temperature 10 °C above the gel–liquid–crystalline phase-transition temperature of the liposomes. The final concentration of lipids was between 10 and 30 mg/mL in the phosphate buffer. The liposomal suspensions were treated ten times with a heating–cooling process in which the liposomes were incubated for 10 min at 4 °C, then heated to a temperature 10 °C higher than the T_m_ of the doped liposomes, and incubated at this temperature, also for 10 min. The liposome morphology was standardized using sonication. Lipid dispersion was ultrasonicated using InterSonic water bath sonicator (Intersonic Sysytems, Poland) at 80 kHz for 30 min. at 55 °C.

#### PEGylated liposomes

A chloroform–methanol solution of DPPC, PEG2000 PE and stigmasterol or its esters was dried under a stream of nitrogen^53^. The dry lipids films were hydrated with the addition of 1 mL of buffer at a temperature 10 °C above the gel–liquid–crystalline phase-transition temperature of the liposomes. The final concentration of lipids was between 10 and 30 mg/mL in the phosphate buffer. The liposomal suspensions were treated ten times with a heating–cooling process in which the liposomes were incubated for 10 min at 4 °C, then heated to a temperature 10 °C higher than the T_m_ of the doped liposomes, and incubated at this temperature, also for 10 min. The liposome morphology was standardized using sonication. Lipid dispersion was ultrasonicated using InterSonic water bath sonicator (Intersonic Sysytems, Poland) at 80 kHz for 30 min. at 55 °C.

### Freeze-drying of liposomes

The liposomes were dried using an Alpha 2–4 LD freeze-dryer (Christ, Germany) with a freeze temperature of -18 °C and a drying temperature of -20 °C for 20 h. Desiccation was then performed at 5 °C for 4 h.

### Digestion of liposomes

Simulated gastric fluid (SGF) and simulated intestinal fluid (SIF) digestions were prepared following Minekus et al.^54^ The lyophilized liposomes were dissolved in 1 mL of ultrapure water. Then 7.5 mL SGF, 75 mg of liposomes, 1.6 mL of pepsin solution (25,000 U/ml to obtain a final concentration of 2,000 U/ml gastric contents) and 5 µl 0.3 M calcium chloride were added and mixed manually for one minute. The pH of the mixture was adjusted to 3 and ultrapure water was added to make up a volume of 20 ml. The gastric mixture was placed back in the shaking bath for two hours under the same conditions. Samples were taken.

To simulate intestinal conditions, 11 ml of SIF, 5 ml of lipase from porcine pancreas solution (800 U/ml based on trypsin activity to obtain a final concentration of 100 U/ml intestinal contents), 40 µl of 0.3 M calcium chloride, and 2.5 ml of bovine bile extract solution (166 or 280 mM bile salts, to obtain a final concentration of 10 or 17.5 mM in intestinal contents) were added. The final mixture was mixed manually for one minute, adjusted to pH 7, and ultrapure water was added to make up a final volume of 40 ml. Finally, this was incubated in a shaking bath for 2 h at 37 °C and 95 rpm. Samples were taken. Each digestion process was performed in duplicate. The digestive tract model is shown in Fig. 1.

### Sterols

The stigmasterol content was determined using AOCS Official Method Ch 6–91^55^. Liposomes were dissolved in chloroform to release the encapsulated substances. The solvent was then evaporated under nitrogen steam and 1 mg of encapsulated substances was saponified. The unsaponifiable fraction was extracted using hexane: methyl *tert* butyl ether (1:1, v/v). After evaporation, the solvent samples were derivatized and the sterols were identified on a gas chromatograph HP 6890 equipped with a DB-35MS capillary column (25 m × 0.20 mm × 0.33 μm; J&W Scientific). Samples were injected at 290 °C in splitless mode. The column temperature was programmed from 100 °C held for five minutes, then increased at a rate of 25 °C/min to 250 °C, where it was held for one minute, and increased at a rate of 3 °C/min to 290 °C, and held for twenty min. The detector temperature was set to 300 °C. Hydrogen was used as a carrier gas at a flow rate of 1.5 ml/min. 5α-Cholestane was used as an internal standard. Stigmasterol was quantified with a general relative response factor of 1.00, which was confirmed using commercially available stigmasterol standards. Stigmasterol was identified by comparison with the retention data of the standard.

### Oxyphytosterols

To determine the derivatives of oxidized phytosterols, the method described by Rudzińska et al. was used^31^. The lipid fraction was extracted using the Folch method with the addition of 0.006% BHT and then transesterified with sodium methoxide. After extraction with chloroform, the sample was fractionated on SEP-PAK NH2 columns, silylated and analyzed by gas chromatography using a Trace 2000 apparatus coupled to a Finnigan - Polaris Q mass spectrometer. The analysis was carried out on a DB-35MS capillary column (25 m × 0.20 mm, 0.33 μm; J&W Scientific, Folsom, California, USA) at programmed temperature: 50–270 °C rise 25 °C/min, 270–290 °C rise 1 °C/min, 290 °C was maintained for 95 min. 20 µg of 19-hydroxy-cholesterol was added to the sample as an internal standard. 19-hydroxy-cholesterol was added to the sample as an internal standard in the amount of 20 µg.

### Cytotoxicity

The cytotoxicity of conventional and PEGylated liposomes encapsulated with stigmasterol and its esters with myristic and oleic acids before and after their gastrointestinal digestion (both gastric and intestine stages) was assessed using human normal small intestinal HIEC-6 (ATCC CRL-3266) and colon mucosa CCD 841CoN (ATCC CRL-179) cells obtained from the American Type Culture Collection (ATCC, Manassas, VA, USA). The cell lines were cultured under the conditions recommended by ATCC. In the cytotoxicity experiments, the cells were grown in 96-well plates at an initial density of 1.5 × 10^4^ cells/cm^2^. After 24 h, cell cultures were treated with liposomes encapsulated with stigmasterol and its esters for 48 h under standard culture conditions. Cell viability and metabolic activity were assessed using the MTT test following the procedure described^56^.

### Statistical analysis

The experiments and analysis were performed in two independent replicates and the data presented are the mean values with standard deviation (± SD). Statistical analysis was performed using the STATISTICA version 13.3 software (Statsoft, Inc., Tulsa, OK, USA). The significance of the main effects was determined by One-way analysis of variance (ANOVA). The equality of variances assumption was verified with Levene’s test. Parametric Tukey’s post hoc test was employed to analyze differences between the mean values of multiple groups. Statistical significance was considered at *p* < 0.05. RStudio (version 2022.07.01 + 554 with packages FactoMineR v.2.4 and factoextra v.1.0.7) was the software used for principal components analysis (PCA).

## Conclusions

Conventional and PEGylated liposomes encapsulated with stigmasterol, stigmasterol myristate and stigmasterol oleate were prepared using DPPC as phospholipid source. The effects of liposome PEGylation, of the choice of encapsulation compound, and of in vitro digestion on the stability of stigmasterol and cytotoxicity were analyzed. Our results show that PEGylation of liposomes and the chemical structure of the encapsulated compounds affect the stability and cytotoxicity of the liposomes following gastrointestinal digestion. Esterification of stigmasterol with fatty acids affects the release of stigmasterol encapsulated in liposomes, especially from PEGylated liposomes. The level of fatty acid saturation has no significant effect on this release. PEGylation of liposomes did not inhibit sterol oxidation, but in fact can induce the formation of SOPs.

The cytotoxicity of conventional liposomes can be ranked in the following order L-St < L-OE < L-ME, but the PEGylation of L-St significantly enhanced their cytotoxic activity. In contrast, PEGylation of liposomes encapsulated with stigmasterol esters (PegL-ME and PegL-OE) did not affect their cytotoxic potential.

Gastrointestinal digestion increased the cytotoxicity of L-St. In contrast, the cytotoxic potential of GID liposomes encapsulated with stigmasterol esters (GID L-ME and GID L-OE) was lower than that of undigested liposomes (L-ME and L-OE). The cytotoxicity to small intestinal cells was as follows: L-St = L-OE < L-ME, while the cytotoxicity to colon mucosal cells was slightly different: L-OE < L-St < GID L-ME. PEGylation of liposomes did not cause an increase in cytotoxicity to the small intestinal cells or colon mucosa cells. However, it should be highlighted that the cytotoxic effects of GID PEGylated liposomes were considerably higher in colon CCD 841CoN cells than in small intestinal HIEC-6 cells.

The use or nonuse of additional substances such as polyethylene glycol (PEG) in liposomes and the esterification of stigmasterol with saturated or unsaturated fatty acids both have significant impact on the stability of the encapsulated substances during gastrointestinal digestion and their cytotoxicity on cells in different parts of digestion tract.

The use of liposomes as carriers of plant sterols in foods still requires much research, including on their bioaccessibility and bioavailability.

## Data Availability

The datasets used and analysed in this are available from https://repod.icm.edu.pl/dataset.xhtml?persistentId=doi:10.18150/UYMIID.

## Abbreviations

PS: Phytosterols, plant sterols
DPPC: Dipalmitoylphosphatidylcholine
St: Stigmasterol
PegL: PEGylated liposomes
L-St: Liposomes encapsulated with free stigmasterol
L-ME: Liposomes encapsulated with stigmasterol myristate
L-OE: Liposomes encapsulated with stigmasterol oleate
7αOHSt: 7α-hydroxystigmasterol
7βOHSt: 7β-hydroxystigmasterol
α-epoxySt: 5α,6α-epoxystigmasterol
β-epoxySt: 5β,6β-epoxystigmasterol
7ketoSt: 7ketostigmasterol
triolSt: 3β,5α,6β-trihydroxystigmasterol
7αOHC: 7α-hydroxycholesterol
7βOHC: 7β-hydroxycholesterol
α-epoxyC: 5α,6α-epoxycholesterol
β-epoxyC: 5β,6β-epoxycholesterol
7ketoC: 7ketocholesterol
triolC: 3β,5α,6β-trihydroxycholesterol

## References

[1] Woyengo, T. A., Ramprasath, V. R. & Jones, P. J. H. Anticancer effects of phytosterols. *Eur. J. Clin. Nutr.* **63**, 813–820 (2009). 19491917 10.1038/ejcn.2009.29

[2] Awad, A. B., Smith, A. J. & Fink, C. S. Plant sterols regulate rat vascular smooth muscle cell growth and Prostacyclin release in culture. *Prostaglandins Leukot. Essent. Fat. Acids*. **64**, 323–330 (2001). 10.1054/plef.2001.0273 11427042

[3] Ramprasath, V. R. & Awad, A. B. Role of phytosterols in cancer prevention and treatment. *J. AOAC Int.* **98**, 735–738 (2015). 26086253 10.5740/jaoacint.SGERamprasath

[4] Feng, S. et al. Intake of stigmasterol and β-sitosterol alters lipid metabolism and alleviates NAFLD in mice fed a high-fat western-style diet. *Biochim. Biophys. Acta - Mol. Cell. Biol. Lipids*. **1863**, 1274–1284 (2018). 30305244 10.1016/j.bbalip.2018.08.004 PMC6226309

[5] Feng, S. et al. Effects of stigmasterol and β-Sitosterol on nonalcoholic fatty liver disease in a mouse model: A lipidomic analysis. *J. Agric. Food Chem.* **66**, 3417–3425 (2018). 29583004 10.1021/acs.jafc.7b06146

[6] Laos, S. et al. Long-term intake of Soyabean phytosterols lowers serum TAG and NEFA concentrations, increases bile acid synthesis and protects against fatty liver development in dyslipidaemic hamsters. *Br. J. Nutr.* **112**, 663–673 (2014). 24932972 10.1017/S0007114514001342

[7] Kim, K. S. et al. Effects of β-sitosterol derived from Artemisia capillaris on the activated human hepatic stellate cells and dimethylnitrosamine-induced mouse liver fibrosis. *BMC Complement. Altern. Med.* **14**, 1–10 (2014). 25262005 10.1186/1472-6882-14-363 PMC4193130

[8] Ostlund, R. E. et al. Gastrointestinal absorption and plasma kinetics of soy ∆5-phytosterols and phytostanols in humans. *Am J. Physiol. - Endocrinol. Metab* **282**, e911–6 (2002). 10.1152/ajpendo.00328.2001 11882512

[9] Ostlund, R. E. Phytosterols, cholesterol absorption and healthy diets. *Lipids* **42**, 41–45 (2007). 17393209 10.1007/s11745-006-3001-9

[10] Kritchevsky, D. & Chen, S. C. Phytosterols-health benefits and potential concerns: A review. *Nutr. Res.* **25**, 413–428 (2005).

[11] Luo, X., Su, P. & Zhang, W. Advances in microalgae-derived phytosterols for functional food and pharmaceutical applications. *Mar. Drugs*. **13**, 4231–4254 (2015). 26184233 10.3390/md13074231 PMC4515614

[12] Hovenkamp, E. et al. Biological effects of oxidized phytosterols: A review of the current knowledge. *Prog Lipid Res.* **47**, 37–49 (2008). 18022398 10.1016/j.plipres.2007.10.001

[13] Tomoyori, H. et al. Phytosterol oxidation products are absorbed in the intestinal lymphatics in rats but do not accelerate atherosclerosis in Apolipoprotein E-deficient mice (Journal of nutrition (2004) 134 (1690–1696)). *J. Nutr.* **134**, 2738 (2004). Erratum. 10.1093/jn/134.7.1690 15226455

[14] Kasprzak, M. et al. The degradation of bioactive compounds and formation of their oxidation derivatives in refined rapeseed oil during heating in model system. *Lwt* **123**, 1090708 (2020).

[15] Kmiecik, D. et al. Stabilisation of phytosterols by natural and synthetic antioxidants in high temperature conditions. *Food Chem.* **173**, 966–971 (2015). 25466113 10.1016/j.foodchem.2014.10.074

[16] Lambelet, P. et al. Formation of modified fatty acids and oxyphytosterols during refining of low erucic acid rapeseed oil. *J. Agric. Food Chem.* **51**, 4284–4290 (2003). 12848499 10.1021/jf030091u

[17] Raczyk, M., Bonte, A., Matthäus, B. & Rudzińska, M. Impact of added phytosteryl/phytostanyl fatty acid esters on chemical parameters of margarines upon heating and Pan-Frying. *Eur. J. Lipid Sci. Technol.* **120**, 1–11 (2018).

[18] Rudzińska, M., Korczak, J. & Wąsowicz, E. Changes in phytosterols and their oxidation products during frying of French Fries in rapeseed oil. *Pol. J. Food Nutr. Sci.* **14**, 381–387 (2005).

[19] Plat, J. et al. Oxidised plant sterols as well as oxycholesterol increase the proportion of severe atherosclerotic lesions in female LDL receptor+/- mice. *Br. J. Nutr.* **111**, 64–70 (2014). 23773414 10.1017/S0007114513002018

[20] Luister, A. et al. Increased plant sterol deposition in vascular tissue characterizes patients with severe aortic stenosis and concomitant coronary artery disease. *Steroids* **99**, 272–280 (2015). 25814070 10.1016/j.steroids.2015.03.011

[21] Baumgartner, S. et al. Effects of plant Stanol ester consumption on fasting plasma oxy(phyto)sterol concentrations as related to fecal microbiota characteristics. *J. Steroid Biochem. Mol. Biol.* **169**, 46–53 (2017). 26940357 10.1016/j.jsbmb.2016.02.029

[22] Adcox, C., Boyd, L., Oehrl, L., Allen, J. & Fenner, G. Comparative effects of phytosterol oxides and cholesterol oxides in cultured macrophage-derived cell lines. *J. Agric. Food Chem.* **49**, 2090–2095 (2001). 11308372 10.1021/jf001175v

[23] Vejux, A. et al. Absence of oxysterol-like side effects in human monocytic cells treated with phytosterols and oxyphytosterols. *J. Agric. Food Chem.* **60**, 4060–4066 (2012). 22490085 10.1021/jf300487r

[24] Alemany, L., Laparra, J. M., Barberá, R. & Alegría, A. Relative expression of cholesterol transport-related proteins and inflammation markers through the induction of 7-ketosterol-mediated stress in Caco-2 cells. *Food Chem. Toxicol.* **56**, 247–253 (2013). 23454145 10.1016/j.fct.2013.02.040

[25] Yang, C. et al. β-Sitosterol oxidation products attenuate vasorelaxation by increasing reactive oxygen species and cyclooxygenase-2. *Cardiovasc. Res.* **97**, 520–532 (2013). 23250922 10.1093/cvr/cvs370

[26] O’Callaghan, Y. et al. Synthesis and assessment of the relative toxicity of the oxidised derivatives of Campesterol and Dihydrobrassicasterol in U937 and HepG2 cells. *Biochimie* **95**, 496–503 (2013). 22561884 10.1016/j.biochi.2012.04.019

[27] He, H. et al. Adapting liposomes for oral drug delivery. *Acta Pharm. Sin B*. **9**, 36–48 (2019). 30766776 10.1016/j.apsb.2018.06.005 PMC6362257

[28] Cui, M. et al. Liposomes containing cholesterol analogues of botanical origin as drug delivery systems to enhance the oral absorption of insulin. *Int. J. Pharm.* **489**, 277–284 (2015). 25957702 10.1016/j.ijpharm.2015.05.006

[29] Peng, T., Xu, W., Li, Q., Ding, Y. & Huang, Y. Pharmaceutical liposomal delivery—specific considerations of innovation and challenges. *Biomater. Sci.* **11**, 62–75 (2022). 36373563 10.1039/d2bm01252a

[30] Emami, S., Azadmard-Damirchi, S., Peighambardoust, S. H., Valizadeh, H. & Hesari, J. Liposomes as carrier vehicles for functional compounds in food sector. *J. Exp. Nanosci.* **11**, 737–759 (2016).

[31] Rudzińska, M. et al. Stigmasterol and its esters encapsulated in liposomes: characterization, stability, and derivative formation. *Food Chem.* **465**, 142039 (2025). 39561597 10.1016/j.foodchem.2024.142039

[32] Vanmierlo, T., Husche, C., Schött, H. F., Pettersson, H. & Lütjohann, D. Plant sterol oxidation products-Analogs to cholesterol oxidation products from plant origin? *Biochimie* **95**, 464–472 (2013). 23009926 10.1016/j.biochi.2012.09.021

[33] Lin, Y. et al. Formation of plant sterol oxidation products in foods during baking and cooking using margarine without and with added plant sterol esters. *J. Agric. Food Chem.* **64**, 653–662 (2016). 26697919 10.1021/acs.jafc.5b04952

[34] Milla, P., Dosio, F. & Cattel, L. PEGylation of proteins and liposomes: a powerful and flexible strategy to improve the drug delivery. *Curr. Drug Metab.* **13**, 105–119 (2011). 10.2174/138920012798356934 21892917

[35] Hatakeyama, H., Akita, H. & Harashima, H. The polyethyleneglycol dilemma: advantage and disadvantage of pegylation of liposomes for systemic genes and nucleic acids delivery to tumors. *Biol. Pharm. Bull.* **36**, 892–899 (2013). 23727912 10.1248/bpb.b13-00059

[36] Mohamed, M., Alaaeldin, E., Hussein, A. & Sarhan, A. Liposomes and pegylated liposomes as drug delivery system. *J. Adv. Biomed. Pharm. Sci.* **0**, 0–0 (2020).

[37] Liu, Y., Liu, D., Zhu, L., Gan, Q. & Le, X. Temperature-dependent structure stability and in vitro release of chitosan-coated Curcumin liposome. *Food Res. Int.* **74**, 97–105 (2015). 28412008 10.1016/j.foodres.2015.04.024

[38] Liu, W. et al. Research progress on liposomes: application in food, digestion behavior and absorption mechanism. *Trends Food Sci. Technol.* **104**, 177–189 (2020).

[39] Liu, W., Ye, A., Liu, C., Liu, W. & Singh, H. Structure and integrity of liposomes prepared from milk- or soybean-derived phospholipids during in vitro digestion. *Food Res. Int.* **48**, 499–506 (2012).

[40] Briuglia, M. L., Rotella, C., McFarlane, A. & Lamprou, D. A. Influence of cholesterol on liposome stability and on in vitro drug release. *Drug Deliv Transl Res.* **5**, 231–242 (2015). 25787731 10.1007/s13346-015-0220-8

[41] Lombardo, D. & Kiselev, M. A. Methods of Liposomes Preparation: Formation and Control Factors of Versatile Nanocarriers for Biomedical and Nanomedicine Application. *Pharmaceutics* 14, (2022). 10.3390/pharmaceutics14030543 PMC8955843 35335920

[42] Song, F., Yang, G., Wang, Y. & Tian, S. Effect of phospholipids on membrane characteristics and storage stability of liposomes. *Innov. Food Sci. Emerg. Technol.* **81**, 103155 (2022).

[43] Tsermentseli, S. K., Kontogiannopoulos, K. N., Papageorgiou, V. P. & Assimopoulou, A. N. Comparative study of pEgylated and conventional liposomes as carriers for Shikonin. *Fluids* **3**, 1–16 (2018).

[44] Wang, X. et al. Are pegylated liposomes better than conventional liposomes? A special case for vincristine. *Drug Deliv*. **23**, 1092–1100 (2016). 26024386 10.3109/10717544.2015.1027015

[45] Maeda, H., Wu, J., Sawa, T., Matsumura, Y. & Hori, K. Tumor vascular permeability and the EPR effect in macromolecular therapeutics: A review. *J. Control Release*. **65**, 271–284 (2000). 10699287 10.1016/s0168-3659(99)00248-5

[46] Alemany, L., Laparra, J. M., Barberá, R. & Alegría, A. Evaluation of the cytotoxic effect of 7keto-stigmasterol and 7keto-cholesterol in human intestinal (Caco-2) cells. *Food Chem. Toxicol.* **50**, 3106–3113 (2012). 22750387 10.1016/j.fct.2012.06.036

[47] Laparra, J. M., Alfonso-García, A., Alegría, A., Barberá, R. & Cilla A. 7keto-stigmasterol and 7keto-cholesterol induce differential proteome changes to intestinal epitelial (Caco-2) cells. *Food Chem. Toxicol.* **84**, 29–36 (2015). 26140950 10.1016/j.fct.2015.06.021

[48] Freitas, F. A., De, Levy, D., Zarrouk, A., Lizard, G. & Bydlowski, S. P. Impact of oxysterols on cell death, proliferation, and differentiation induction: current status. *Cells* **10**, 2301 (2021). 34571949 10.3390/cells10092301 PMC8468221

[49] Iwanaga, K. et al. Oral delivery of insulin by using surface coating liposomes. Improvement of stability of insulin in GI tract. *Int. J. Pharm.* **157**, 73–80 (1997).

[50] Li, H., Song, J. H., Park, J. S. & Han, K. Polyethylene glycol-coated liposomes for oral delivery of Recombinant human epidermal growth factor. *Int. J. Pharm.* **258**, 11–19 (2003). 12753749 10.1016/s0378-5173(03)00158-3

[51] Ma, Q. et al. Oral absorption enhancement of probucol by pegylated G5 PAMAM dendrimer modified nanoliposomes. *Mol. Pharm.* **12**, 665–674 (2015). 25587935 10.1021/mp500388m PMC4770526

[52] Cieślik-Boczula, K., Szwed, J., Jaszczyszyn, A., Gasiorowski, K. & Koll, A. Interactions of dihydrochloride fluphenazine with DPPC liposomes: ATR-IR and31P NMR studies. *J. Phys. Chem. B*. **113**, 15495–15502 (2009). 19883091 10.1021/jp904805t

[53] Cieślik-Boczula, K., Küpcü, S., Rünzler, D., Koll, A. & Köhler, G. Effects of the phenolic lipid 3-pentadecylphenol on phospholipid bilayer organization. *J. Mol. Struct.* **919**, 373–380 (2009).

[54] Minekus, M. et al. A standardised static in vitro digestion method suitable for food-an international consensus. *Food Funct.* **5**, 1113–1124 (2014). 24803111 10.1039/c3fo60702j

[55] AOCS Official Method Ch 6–91. Determination of the composition of the sterol fraction of animal and vegetable oils and fats by TLC and capillary GLC. *Off Methods Recomm Pract. Am. Oil Chem. Soc* 4–7 (2009).

[56] Olejnik, A. et al. Gastrointestinal digested Sambucus Nigra L. fruit extract protects in vitro cultured human colon cells against oxidative stress. *Food Chem.* **197**, 648–657 (2016). 26616999 10.1016/j.foodchem.2015.11.017

